# A Peptide Derived from the HIV-1 gp120 Coreceptor-Binding Region Promotes Formation of PAP248-286 Amyloid Fibrils to Enhance HIV-1 Infection

**DOI:** 10.1371/journal.pone.0144522

**Published:** 2015-12-14

**Authors:** Jinquan Chen, Ruxia Ren, Suiyi Tan, Wanyue Zhang, Xuanxuan Zhang, Fei Yu, Tianrong Xun, Shibo Jiang, Shuwen Liu, Lin Li

**Affiliations:** 1 School of Pharmaceutical Sciences, Southern Medical University, Guangzhou, China; 2 Key Laboratory of Medical Molecular Virology of MOE/MOH, Shanghai Medical College and Institute of Medical Microbiology, Fudan University, Shanghai, China; 3 Lindsley F. Kimball Research Institute, New York Blood Center, New York, NY, United States of America; University of Maryland School of Medicine, UNITED STATES

## Abstract

**Background:**

Semen is a major vehicle for HIV transmission. Prostatic acid phosphatase (PAP) fragments, such as PAP248-286, in human semen can form amyloid fibrils to enhance HIV infection. Other endogenous or exogenous factors present during sexual intercourse have also been reported to promote the formation of seminal amyloid fibrils.

**Methodology and Principal Findings:**

Here, we demonstrated that a synthetic 15-residue peptide derived from the HIV-1 gp120 coreceptor-binding region, designated enhancing peptide 2 (EP2), can rapidly self-assemble into nanofibers. These EP2-derivated nanofibers promptly accelerated the formation of semen amyloid fibrils by PAP248-286, as shown by Thioflavin T (ThT) and Congo red assays. The amyloid fibrils presented similar morphology, assessed via transmission electron microscopy (TEM), in the presence or absence of EP2. Circular dichroism (CD) spectroscopy revealed that EP2 accelerates PAP248-286 amyloid fibril formation by promoting the structural transition of PAP248-286 from a random coil into a cross-β-sheet. Newly formed semen amyloid fibrils effectively enhanced HIV-1 infection in TZM-bl cells and U87 cells by promoting the binding of HIV-1 virions to target cells.

**Conclusions and Significance:**

Nanofibers composed of EP2 promote the formation of PAP248-286 amyloid fibrils and enhance HIV-1 infection.

## Introduction

In 2013, an estimated 35 million people were living with human immunodeficiency virus (HIV) worldwide, and approximately 2.1 million people were newly infected with HIV [1]. Sexual transmission of HIV, including both heterosexual and homosexual transmission, is responsible for the majority of HIV infections in many developing countries. Identification of the host and viral factors that significantly enhance HIV infection is critical for developing strategies to prevent sexual transmission of HIV [2–4].

Semen acts as a vector for HIV transmission through sexual intercourse and plays an important role in the spread of HIV/AIDS [5]. Semen harbors several crucial biological factors that may affect the spread of HIV [6–8]. Notably, semen boosts HIV infectivity and impairs the antiviral efficacy of microbicides [9]. Seminal amyloid fibrils have been shown to enhance HIV infectivity. One of the best-characterized seminal amyloid fibrils is SEVI (semen-derived enhancer of virus infection). SEVI fibrils are formed by a peptide derived from residues 248 to 286 of prostatic acid phosphatase (PAP). This peptide, designated PAP248-286, can reportedly enhance the infectious titer of HIV-1 by up to five orders of magnitude [10, 11]. Other PAP fragments (e.g., PAP85-120) and semenogelins (SEM1 and SEM2) also promote HIV-1 infection by forming amyloid fibrils in seminal fluid [12–14]. Seminal plasma or bacterial curli proteins may promote the formation of seminal amyloid fibrils [15, 16]. Collectively, seminal amyloid fibrils are exploited by HIV to promote its infection via sexual transmission.

We previously demonstrated that three peptides, termed enhancing peptides (EPs), derived from the HIV-1_MN_ envelope gp120 glycoprotein blocked T-20-mediated anti-HIV activity [17]. Coincidentally, several short peptides derived from the HIV-1 gp120 and gp41 envelope glycoproteins were found to assemble spontaneously into stable nanofibrils and significantly facilitate HIV infection [18–20]. More recently, our group demonstrated that some EPs can also form amyloid fibrils and are able to enhance HIV-1 infection [21]. However, it is unclear whether these EPs directly enhance HIV-1 infection through the formation of amyloid fibrils or whether other indirect mechanisms of action are responsible. One EP, a 15-residue peptide derived from the HIV-1_MN_ gp120 coreceptor-binding region (EP2, aa 417–431, QCKIKQIINMWQEVG), was found to enhance HIV-1 infection. Gp120 is considered an Ig superantigen (Ig-SAg) [22]. Gp120 residues 421–433 (KQIINMWQEVGK) form a B cell superantigenic (Sag) site on the protein and contain amino acids that are critical for binding to host CD4 receptors. The 421–433 epitope is conserved in simian immunodeficiency virus (SIV) and relatively conserved in diverse HIV strains. Residues 421–433 of gp120 are recognized by immunoglobulins (Igs) and catalyze its hydrolysis through a serine protease-like mechanism in uninfected humans [23]. Conserved sequences exist between EP2 and residues 421–433 of gp120. Notably, the EP2 sequence is found in a short peptide fragment (INMWQG) that is produced by gp120 degradation in native gp120-loaded rat hepatocytes [24]. Therefore, EP2 might be a critical factor in enhancing HIV infection. In this study, we examined the effect of EP2 on the conversion of PAP248-286 into amyloid fibrils and its potential role in enhancing HIV-1 infection.

## Materials and Methods

### Peptides, cell culture, plasmids and reagents

The peptide PAP248-286 was synthesized and purified by GL Biochem (Shanghai, China) into a lyophilized powder. The lyophilized PAP248-286 peptide (>95% purity) was dissolved in phosphate-buffered saline (PBS) to a concentration of 10 mg/ml. EP2 was synthesized and purified by Huada Biotech Company (Shanghai, China). Lyophilized EP2 (>90% purity) was dissolved in dimethylsulfoxide (DMSO) or deionized water to a concentration of 10 mg/ml. Stock solutions of the peptides were divided into aliquots and stored at -20°C. MT-2 cells, TZM-bl cells, U87-CD4-CCR5 cells, the pNL4-3E^-^R^-^Luc plasmid, HIV-1 Env-encoding plasmids and the peGFP-Vpr plasmid were obtained from the National Institutes of Health AIDS Research and Reference Reagent Program. GHOST (3) Hi-5 and HEK-293 T cells were purchased from the ATCC (Manassas, VA). Plasmids encoding CXCR4-tropic HIV-1 NL4-3, CCR5-tropic HIV-1 SF-162, and dual-tropic 81A and NL4-3 infectious clones were kindly provided by Jan Münch of Ulm University (Ulm, Badenwürttemberg, Germany). Polyethyleneimine (PEI), hen egg-white lysozyme (HEWL), bovine insulin, thioflavin T and Congo Red Kits were purchased from Sigma-Aldrich (St. Louis, MO). ProteoStat Amyloid Plaque Detection Kits were purchased from Enzo Life Sciences (Plymouth Meeting, PA).

### Assaying the capacity of EP2 peptides to self-assemble into nanofibers *in vitro*

The ability of EP2 to self-assemble into nanofibers was evaluated by two methods based on ThT fluorescence assays. First, a concentration-dependent experiment was performed. For this assay, 5 μl samples containing graded concentrations of EP2 (2, 1, 0.5, 0.25 and 0.125 mg/ml in PBS) were stained using 195 μl ThT (50 μM in PBS) [19, 25]. Fluorescence intensity was measured using an RF-5301 PC spectrofluorophotometer (Shimadzu) with an excitation wavelength of 440 nm (5 nm bandwidth) and an emission wavelength of 482 nm (10 nm bandwidth). Second, a time-dependent experiment was performed. For this assay, a 5 μl EP2 sample (2 mg/ml in PBS) was added to 195 μl ThT (50 μM in PBS), and ThT fluorescence intensity (averaging over 30 s) was measured every 5 min for 30 min using an RF-5301 PC spectrofluorophotometer. The size and zeta potential of the EP2 formed fibers (1 mg/ml in deionized water) were determined at 25°C using a Zetasizer Nano ZS instrument (Malvern, Worcestershire, UK). Each measurement was performed in triplicate. EP2 fiber morphology at two different final concentrations (300 and 10 μg/ml) at two time points following agitation (1 min and 48 h) was visualized on an H-7650 transmission electron microscope using an accelerating voltage of 80 kV (Hitachi Limited, Tokyo, Japan).

### Observing EP2 and PAP248-286 fibril morphology via TEM

Fibrils were generated by incubating 3 mg/ml PAP248-286 in the presence or absence of EP2 (100 μg/ml) with agitation at 1,400 rpm at 37°C in an Eppendorf Thermomixer (Hamburg, Germany). Fibril morphology was visualized via TEM at different time points following agitation. To accomplish this, PAP248-286 fibers (300 μg/ml) incubated with or without EP2 (10 μg/ml) were collected at different time points following agitation (0, 4, 8, 12, 24 and 48 h). All samples were tenfold diluted in PBS buffer. The samples were then deposited on glow-discharged, carbon-coated grids for 2 min and negatively stained with 2% phosphotungstic acid for another 2 min. Amyloid fibril morphology was visualized as described above.

### Effects of EP2 on PAP248-286 aggregation

To select the optimum concentration of EP2 to foster the aggregation of PAP248-286, graded concentrations of EP2 (0, 25, 100 and 400 μg/ml) were agitated with PAP248-286 (3 mg/ml) at 1,400 rpm at 37°C. After screening, 3 mg/ml PAP248-286 in the presence or absence of EP2 (100 μg/ml) was agitated at 37°C for 48 h at 1,400 rpm. Individual solutions of PAP248-286 and EP2 alone were agitated under the same conditions to serve as negative controls. Aggregation was examined at different time points (0, 2, 4, 6, 8, 12, 24, 36 and 48 h) by ThT and Congo red staining as previously described [19, 25]. For the ThT assay, 5 μl samples were added to 195 μl ThT working solution (50 μM). After mixing, the fluorescence intensity was measured as described above. For the Congo red staining assay, 10 μl samples were added to 200 μl of Congo red solution from a Congo Red Kit (Sigma). After incubating for 2 min at room temperature, the mixtures were centrifuged at 12,000 rpm for 5 min, and the red-dyed fiber precipitate was dissolved in 50 μl of DMSO. The absorbance was then measured at 490/650 nm using an ELISA reader (Tecan, Research Triangle Park, NC). To analyze the effects of EP2 on the formation of other amyloid fibrils, HEWL and bovine insulin were used as controls [26, 27]. Briefly, sample solutions were prepared by dissolving HEWL in 1 M NaCl solution (final HEWL concentration of 120 μM) with or without EP2 solution (final concentration of 100 μg/ml in deionized water); small amounts of 0.1 M HCl were added to adjust the pH of solution to 4.2. Bovine insulin samples (final concentration of 2 mg/ml) were prepared by diluting a fresh stock solution of 20 mg/ml (3.5 mM) bovine insulin in 0.04 M HCl (pH 1.6) into 20 mM phosphate and 0.1 M NaCl buffer (pH 7.4) with or without EP2 solution (final concentration of EP2 is 100 μg/ml in deionized water) immediately prior to the experiment. The mixtures were agitated at 1,400 rpm at 37°C and examined at different time points via ThT assay [26]. EP2 (100 μg/ml) alone was agitated under the same conditions as a negative control. To assess EP2 fiber stability, we further detected the ThT fluorescence intensity of EP2 fibers (1 mg/ml) in 1 M NaCl solution at different pH values (3.0, 7.4 and 10.0) at varying time points (0, 1, 2, 3, 4, 5, 6, 24 and 48 h).

### CD spectroscopy analysis of fibril secondary structure

PAP248-286 (3 mg/ml) was agitated in the presence or absence of EP2 (100 μg/ml) at 1400 rpm at 37°C as described above. The secondary β-sheet structures of PAP248-286, EP2 and the mixture of PAP248-286 and EP2 were examined at different time points after agitation (0, 8 and 48 h) using CD spectroscopy in the far-UV spectral region between 180 and 260 nm. The formed fibers were diluted 15 times in PBS buffer prior to CD analysis. Therefore, the final concentrations of the PAP248-286 and EP2 fibers were 200 μg/ml and 6.67 μg/ml, respectively. The CD spectra were reported at room temperature using a Jasco 715 spectropolarimeter (Jasco Inc., Japan) equipped with a thermostat-controlled cell housing and cells with a 1-mm path length [17]. Each spectrum was recorded at least three times to ensure the reproducibility of the results. The spectra were corrected via the subtraction of a solvent-only blank. The quantities of amyloid fibrils with different secondary structures were estimated based on the molar residue ellipticity using Jasco software utilities as previously described [17, 28].

### The effects of PAP248-286-formed amyloid fibrils on eGFP-labeled HIV-1 virion binding to target cells

EGFP-labeled HIV-1 virions were produced by PEI-mediated cotransfection of 293T cells with proviral DNA expression plasmids and peGFP-Vpr plasmids as previously described [29]. Culture supernatants were replaced with fresh medium after transfection overnight at 37°C. Two days after transfection, culture supernatants were collected, clarified by sedimentation and stored in aliquots at -80°C for later use.

The binding of eGFP-labeled HIV-1 virions to PAP248-286 amyloid fibrils was examined by fluorescence microscopy. Briefly, 200 μg/ml PAP248-286, 6.67 μg/ml EP2 and mixtures of PAP248-286 and EP2 collected at different time points following agitation (0, 8 and 48 h) were stained with Proteostat dye from a ProteoStat Amyloid Plaque Detection Kit at room temperature as previously described [30]. The samples were incubated 1:1 with eGFP-labeled HIV-1 virions (R5-tropic) at 37°C for 30 min and imaged using a laser scanning Nikon A1 confocal microscope (Nikon, Japan). The effects of PAP248-286-formed amyloid fibrils on the binding of eGFP-labeled HIV-1 virions to target cells were also measured by fluorescence microscopy [11, 13]. First, PAP248-286 and EP2 samples were incubated 1:1 with eGFP-labeled HIV-1 virions at 37°C for 30 min. Then, 3×10^4^/ml GHOST (3) Hi-5 cells were incubated with the pretreated HIV-1 virions at 37°C for another 30 min. The cells were then fixed in paraformaldehyde and stained using a ProteoStat Amyloid Plaque Detection Kit and Hoechst 33342 stain (Enzo Life Sciences) for 30 min at room temperature. Finally, the cells were washed three times with PBS buffer and imaged with a laser scanning confocal microscope (Nikon, Japan).

### Determination of the ability of PAP248-286-formed amyloid fibrils with assistance of EP2 to enhance HIV-1 infection

HIV-1 Env-pseudotyped viruses were produced by cotransfecting cells with pNL4-3E^-^R^-^Luc and the HIV-1 Env-encoding plasmid JR-FL via PEI transfection as previously described [31–33]. To determine the ability of PAP248-286-formed amyloid fibrils (3 mg/ml) to enhance pseudotyped HIV-1 infection in the presence of EP2 (100 μg/ml), U87-CD4-CCR5 cells were seeded into 96-well microtiter plates at 1 × 10^4^ cells/well and incubated at 37°C overnight. Agitated peptide solutions (final PAP248-286 concentration of 30 μg/ml) were incubated with HIV-1 Env-pseudotyped virus for 5 min. Following this, U87-CD4-CCR5 cells were incubated with the peptide-virus mixtures at 37°C for 3 h. To minimize the toxic effects caused by amyloid fibrils, the culture supernatants were replaced with fresh medium after 3 h. The cells were collected 72 h post-infection, and luciferase activity was detected using a luciferase assay kit (Promega, Madison, WI). Furthermore, CXCR4-tropic NL4-3, CCR5-tropic SF-162, and dual-tropic 81A and NL4-3 infectious HIV-1 clones were produced by transfection using proviral DNA expression plasmids and PEI transfection reagent as previously described [10, 11]. To assess HIV-1 virion infectivity in the presence of the agitated peptide solutions, TZM-bl cells at a density of 1 × 10^4^ cells/well were incubated for 3 h with different infectious HIV-1 clones in the absence or presence of agitated peptide solutions at different time points (final PAP248-286 concentration of 30 μg/ml). The culture supernatants were then replaced with fresh medium. At 72 h post-infection, the cells were collected, washed and lysed with a lysing reagent, and luciferase activity was detected using a luciferase assay kit (Promega, Madison, WI). Data were analyzed using SPSS version 19.0 via one-way ANOVA and Dunnett’s post hoc multiple comparisons test (IBM Corporation, Armonk, NY).

## Results

### EP2 peptides rapidly self-assemble into nanofibers in PBS

**Fig 1:**
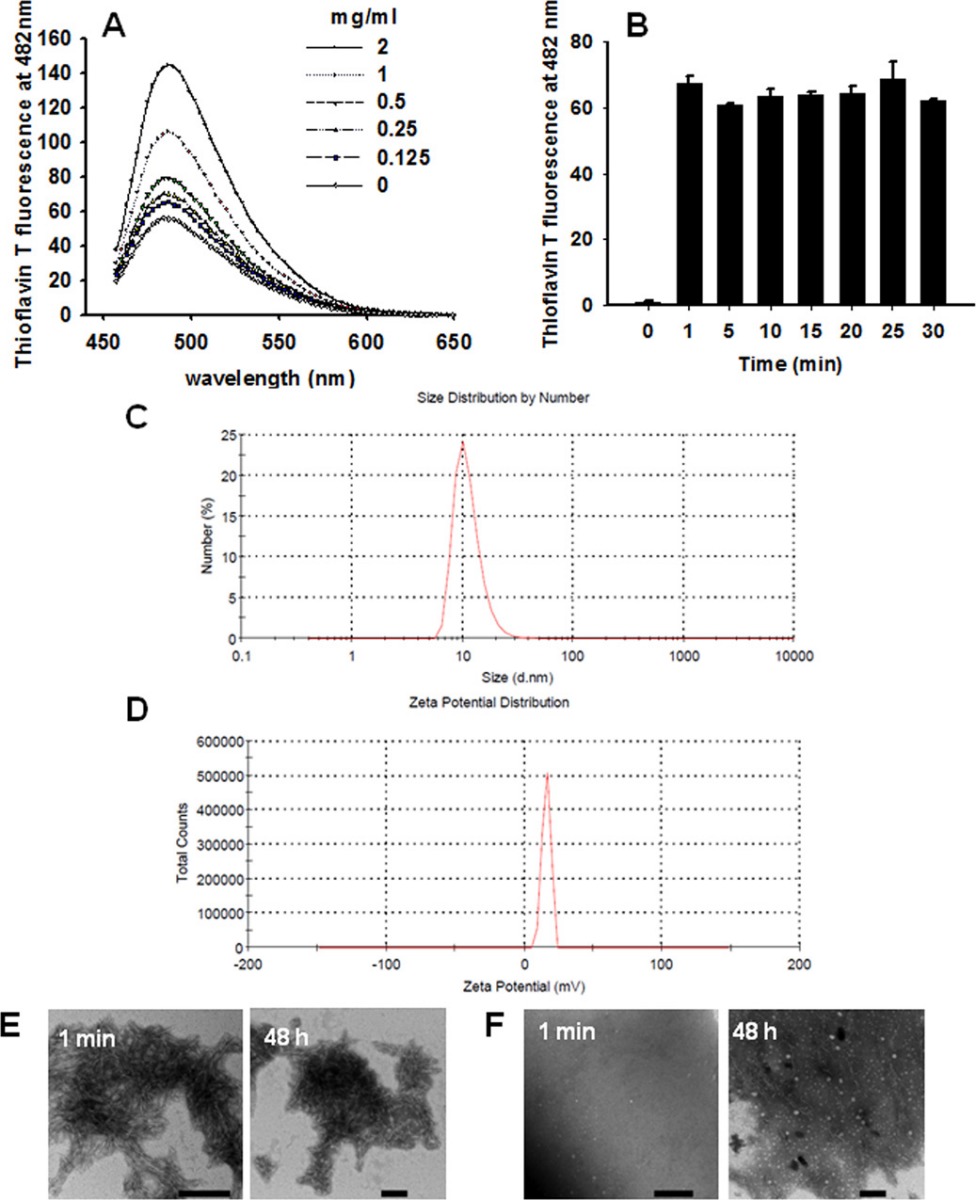
EP2 self-assembles into amyloid fibrils. (A) Different concentrations of EP2 formed amyloid fibrils immediately after gradient dilution, as shown by ThT assay results. (B) EP2 (2 mg/ml) rapidly formed amyloid aggregates, as shown by ThT assay results (every 5 min for 30 min). Readings from a blank control were subtracted from all samples. (C) The mean hydrodynamic size of EP2 fibrils (1 mg/ml). (D) The zeta potential of EP2 fibrils (1 mg/ml). EP2 alone at a (E) final concentration of 300 μg/ml and a (F) final concentration of 10 μg/ml was imaged at two time points (1 min and 48 h) as a negative control. The scale bar is 1 μm.

To evaluate the capacity of EP2 peptides to form fibers and assess the characteristics of such fibers *in vitro*, we diluted a DMSO-containing stock solution of EP2 peptides into PBS. ThT, which is the most commonly used dye for detecting amyloid fibril formation, was used to assess the formation of EP2 nanofibers. The results showed that EP2 could form fibrils almost immediately following dilution into PBS buffer in a concentration-dependent manner at room temperature (Fig 1A). A time-dependent assay showed that a 2 mg/ml EP2 solution rapidly (in 1 min) formed fibers (Fig 1B). The size and the zeta potential of the EP2 fibers were determined using a Zetasizer Nano ZS instrument. Zeta potential is widely used to quantitate the magnitude of a charge. A solution or dispersion with high zeta potential (more than 30 mV) is considered to be electrostatically stabilized, while colloids with low zeta potentials (from 0 to 30 mV) have a tendency to aggregate. The mean hydrodynamic size of the EP2 fibrils was 9.4±1.7 nm (Fig 1C), and their zeta potential was +16.7±1.0 mV (Fig 1D); these values suggest that EP2 forms nanofibers. We next utilized TEM to evaluate EP2 nanofibers formed at a normal concentration (final concentration of 300 μg/ml) and at a very low concentration (final concentration of 10 μg/ml) at 1 min and 48 h following dilution into PBS. For the samples at a normal concentration, branching, needle-like, short fibrils were revealed at both 1 min and 48 h (Fig 1E). For the samples at a low concentration, granular species with no fiber-like structures were observed at 1 min after dilution, and both granular species and small fibers were found at 48 h (Fig 1F). Collectively, these results confirm that the EP2 peptide can rapidly self-assemble into nanofibers *in vitro*.

### EP2 greatly accelerates PAP248-286 amyloid fibril formation

**Fig 2:**
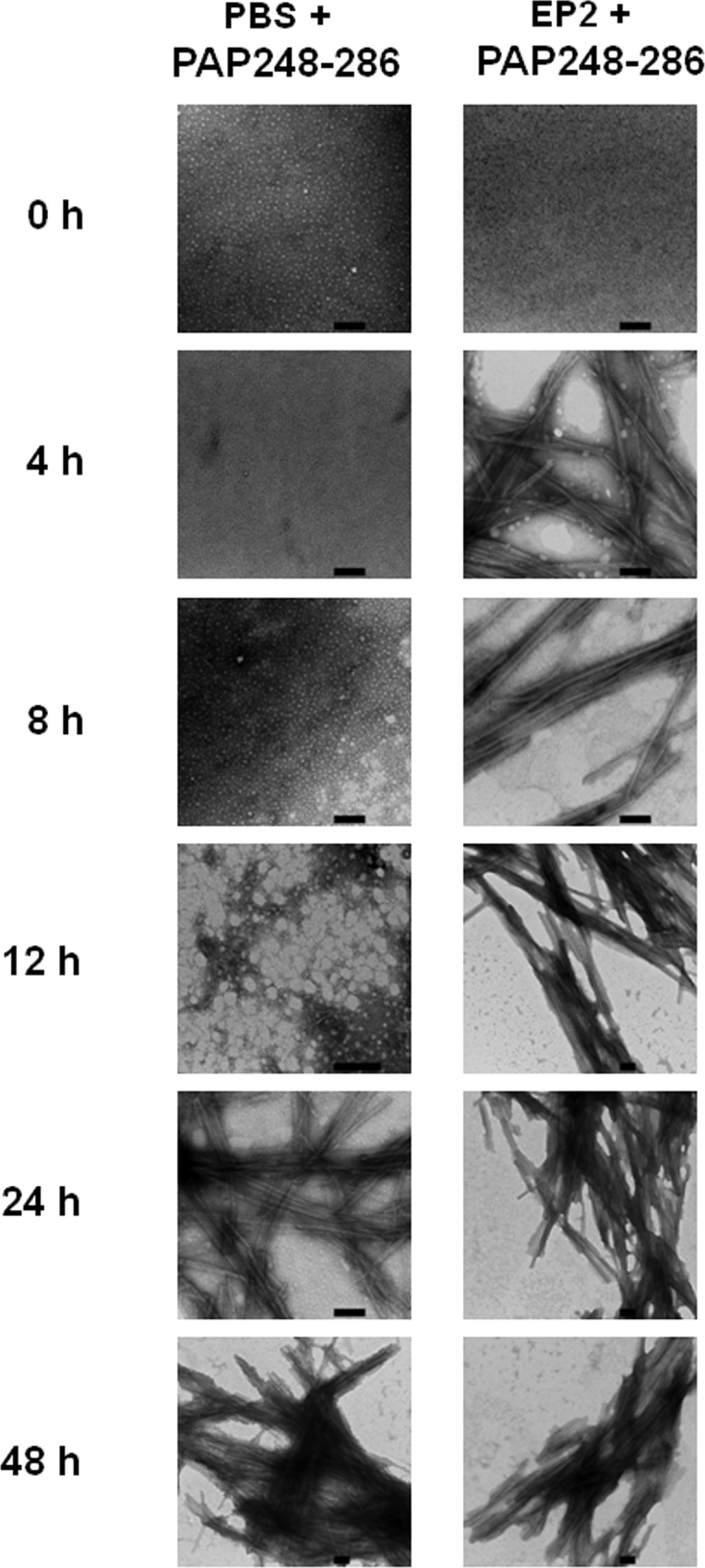
EP2 accelerates PAP248-286 amyloid fibril formation, as shown by TEM. PAP248-286 (3 mg/ml) was agitated to allow fibril formation in the presence or absence of EP2 (100 μg/ml). Amyloid fibril samples (300 μg/ml) collected at different time points following agitation (0, 4, 8, 12, 24 and 48 h) were visualized by negative staining under TEM. The scale bar is 1 μm.

Utilizing a variety of approaches, we next investigated whether EP2-formed nanofibers promote PAP248-286 amyloid fibril formation. Following dilution into PBS, PAP248-286 (3 mg/ml) could not sponentanously form amyloid fibrils at room temperature; this condition was tested for over 72 hours. Thus, unlike EP2, PAP248-286 cannot form fibers under physiological conditions. However, after agitating PAP248-286 at a concentration of 3 mg/ml in PBS at 37°C for 12 h at 1,400 rpm, small fibers were observed under TEM. At 24 h after agitation, branching, needle-like, long amyloid fibrils were revealed, suggesting that PAP248-286 slowly forms amyloid fibrils under agitation (Fig 2).

To determine how EP2 (100 μg/ml) affects PAP248-286 amyloid fibril formation, the presence of amyloid fibrils at different time points after agitation (0, 4, 8, 12, 24 and 48 h) in the presence of EP2 was evaulated by TEM. As shown in Fig 2, in the presence of EP2, PAP248-286 amyloid fibrils formed after shaking for 4 h; in the absence of EP2, fibril formation occurred after shaking for 24 h. These results suggest that EP2 accelarates PAP248-286 amyloid fibril formation.

**Fig 3:**
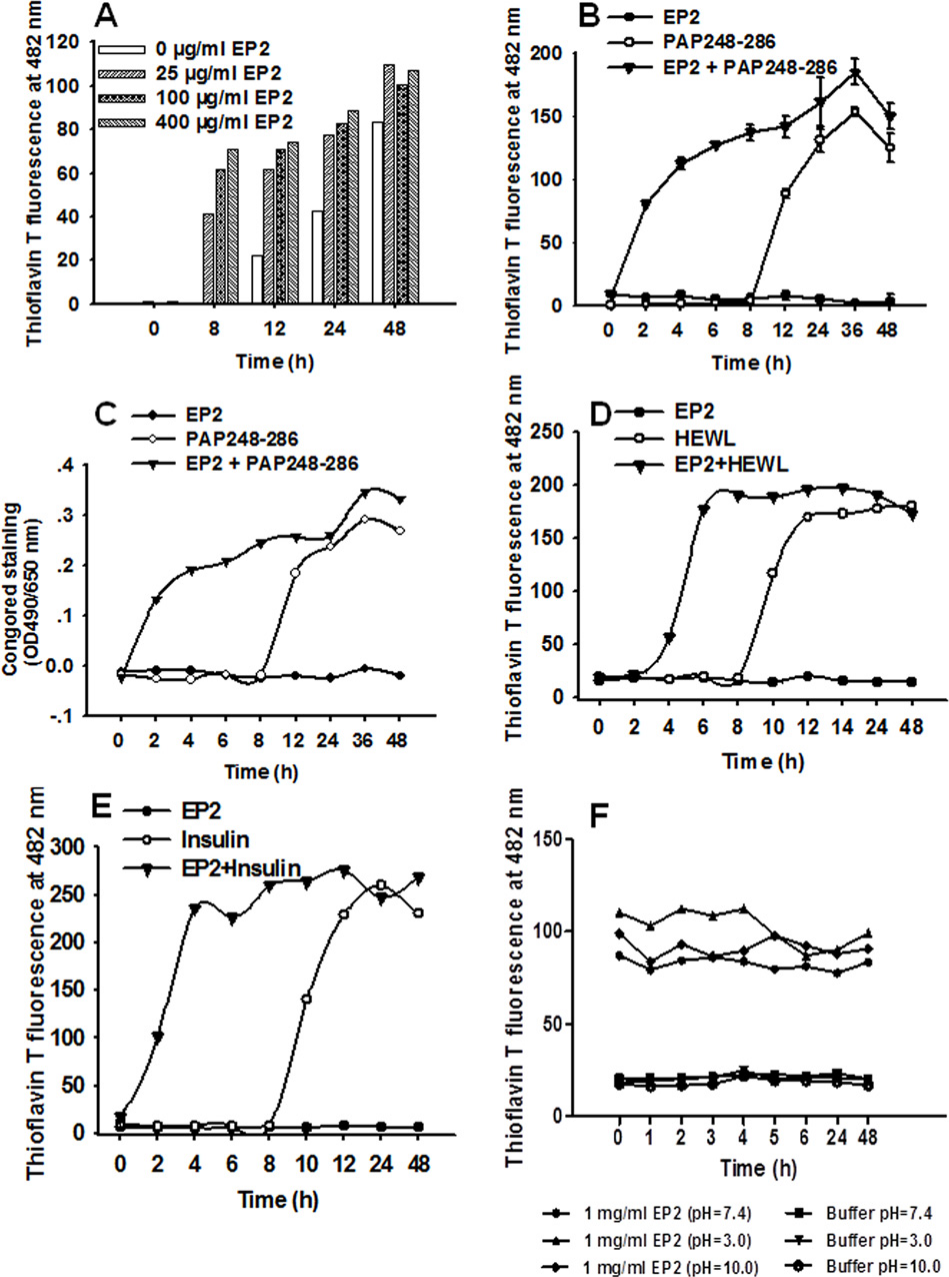
EP2 greatly accelerates PAP248-286 amyloid fibril formation, as shown by ThT assay. (A) EP2 promoted PAP248-286 amyloid fibril formation (3 mg/ml) in a concentration-dependent manner, as shown by ThT assay. EP2 (100 μg/ml) reduced the lag time of amyloid fibril formation, as shown by both ThT fluorescence (B) and Congo red assays (C). (D) EP2 (100 μg/ml) promoted the formation of amyloid fibrils from hen egg-white lysozyme (HEWL, 120 μM), as shown by ThT assay. EP2 alone served as a negative control. Readings for a blank control were subtracted from all samples.

We next measured the kinetics of amyloid formation and PAP248-286 aggregation in the presence or absence of EP2. As shown in Fig 3A, EP2 increased the rate of PAP248-286 amyloid fibril formation after agitation in a concentration-dependent manner, as assessed by ThT fluorescence assay. Moreover, the addition of preformed EP2 fibrils (100 μg/ml) reduced the lag time of PAP248-286 amyloid fibril formation by approximately 2 h, as measured by both ThT fluorescence and Congo red assay (Fig 3B and 3C). In contrast, the lag time of PAP248-286 amyloid fibril formation in the absence of EP2 was approximately 8 to 12 h. EP2 alone did not bind to either ThT or Congo red amyloid-specific dyes at the lowest tested concentration (2.5 μg/ml for the ThT assay and approximately 5 μg/ml for the Congo red assay). Overall, the addition of the EP2 peptide increased the rate of fibrillization. Furthermore, we observed that EP2 promoted the aggregation of HEWL into amyloid fibrils with an approximate lag time of 2 to 4 h, as measured by ThT assay (Fig 3D); the lag time of HEWL fibril formation in the absence of EP2 was approximately 8 to 10 h at pH 4.2 (Fig 3D). We further tested how the addition of EP2 seeds to insulin affects fibril formation at 37°C at neutral pH under stirring. In the presence of EP2 seeds, insulin fibril formation was significantly accelerated (Fig 3E). The lag times of insulin fibril formation with and without EP2 seeds were approximately 2 h and 8 to 10 h, respectively. Stability testing showed that EP2 fibers were fairly stable at different pH values for up to 48 h (Fig 3F). These results further confirm that EP2 nanofibers accelerate the formation of PAP248-286 amyloid fibrils.

### EP2 promotes PAP248-286 beta-sheet formation, as shown by CD spectroscopy

**Fig 4:**
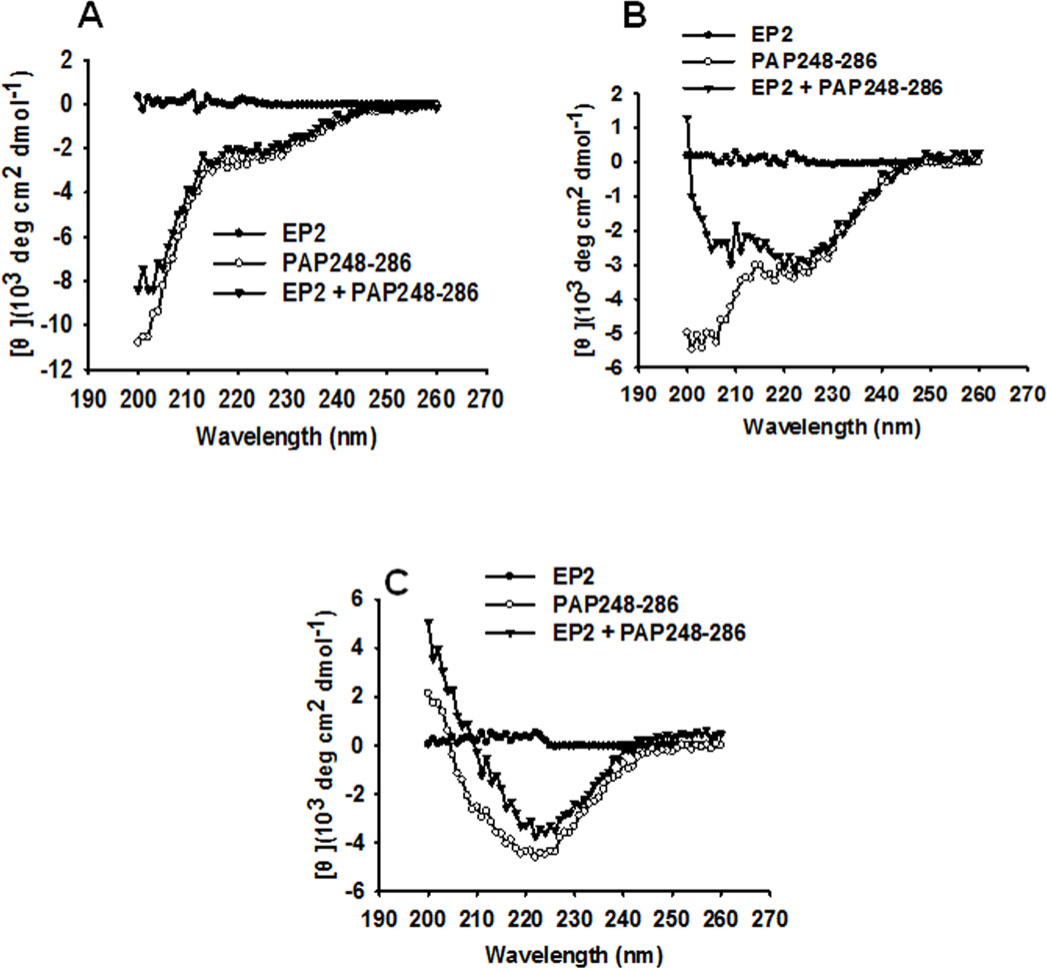
EP2 promoted PAP248-286 to undergo a structural transition to a beta-sheet conformation by CD spectroscopy. A 3 mg/ml sample of PAP248-286 was agitated in the presence or absence of EP2 (100 μg/ml). Sample spectra were collected at the following time points after agitation: (A) 0 h, (B) 8 h and (C) 48 h. Readings from a blank control were subtracted from all samples.

Beta-sheet aggregation is a distinguishing feature of amyloid fibril formation. To determine the effects of EP2 on PAP248-286 β-sheet aggregation, we evaluated the conformational changes associated with PAP248-286 aggregation in the presence or absence of EP2 by characterizing the CD spectra associated with each condition. As shown in Fig 4A, the spectra of PAP248-286 with or without EP2 with no agitation revealed the presence of a characteristic random coiled structure. After shaking for 8 h at 37°C, in the presence of EP2, PAP248-286 underwent an obvious transition from a random coil structure to a β-sheet conformation with a minimum absorbance at 220 nm (Fig 4B). The spectrum of PAP246-248 alone showed the formation of a characteristic random coil structure at 8 h, indicating that β-sheet aggregation had not occurred (Fig 4B). After agitation for 48 h, PAP248-286 displayed a typical β-sheet structure whether in the presence of absence of EP2 (Fig 4C). As shown in Fig 4A–4C, EP2 alone displayed a random coil structure. These results indicate that EP2 accelerates PAP248-286 amyloid fibril formation by promoting the structural transition of PAP248-286 from a random coil to a cross-β-sheet conformation.

### PAP248-286 amyloid fibrils formed in the presence of EP2 enhanced HIV-1 virion binding to target cells

**Fig 5:**
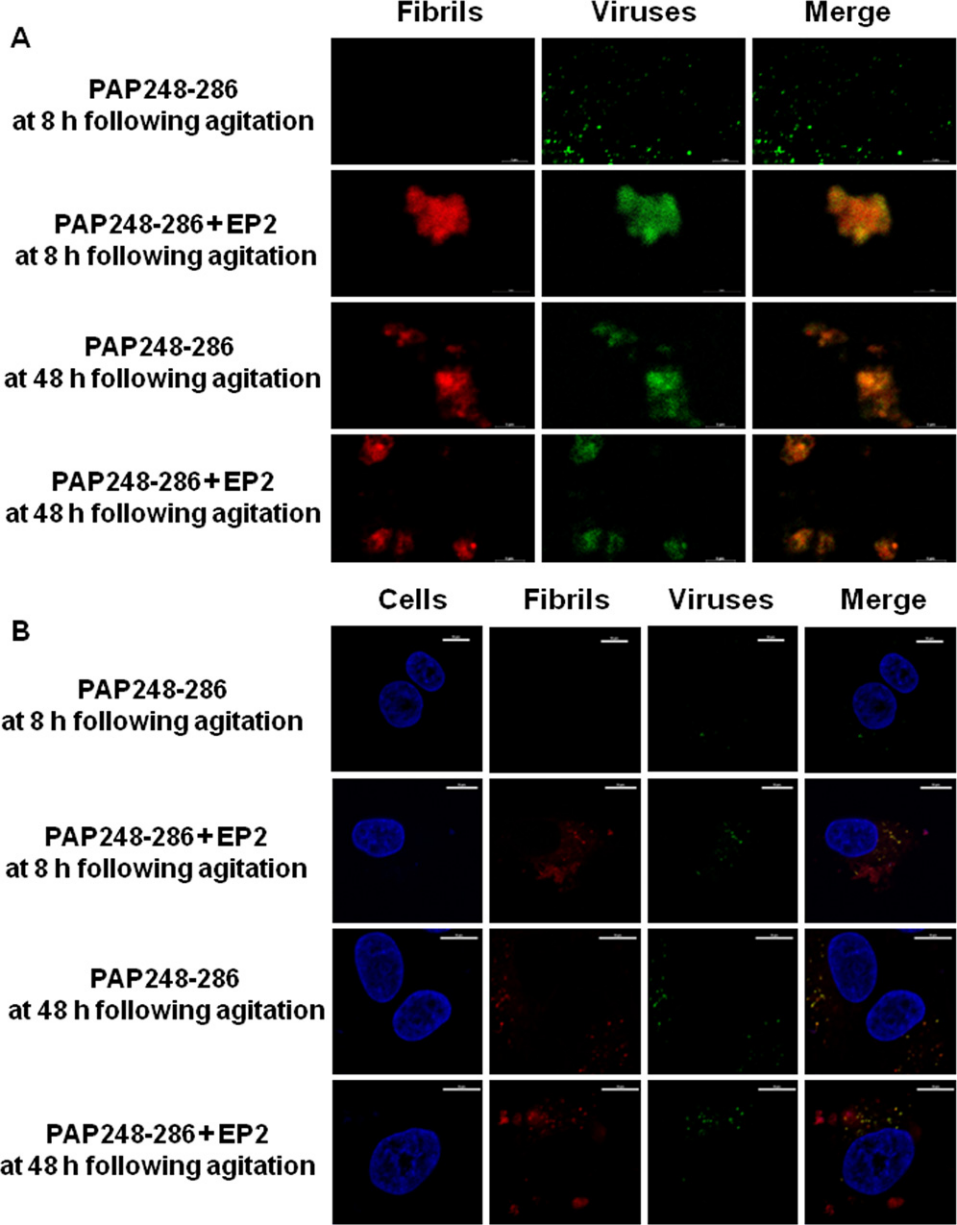
EP2 enhanced PAP248-286 fibril formation and promoted the attachment of HIV-1 virions to target cells. (A) ProteoStat-stained amyloid fibrils at different time points following agitation (100 μg/ml, red) were mixed with eGFP-labeled HIV-1 virions (green, R5 strains) for 3 h, and the samples were imaged using fluorescence confocal microscopy. The scale bar is 5 μm. (B) ProteoStat-stained amyloid fibrils at different time points following agitation (200 μg/ml, red) were added to eGFP-labeled HIV-1 virions (green, R5 strains), and the mixtures were added to GHOST (3) Hi-5 cells (blue). The effects of fibril presence on the binding of HIV-1 virions to target cells were assessed using fluorescence confocal microscopy. The scale bar is 10 μm.

Fluoresence confocal microscopy revealed that HIV-1 virions can bind to PAP248-286 amyloid fibrils (Fig 5A). Amyloid fibrils were stained for visualization using an amyloid-specific red fluorescent dye (ProteoStat Amyloid Plaque Detection Kit). After staining, amyloid fibrils were incubated with eGFP-labeled HIV-1 virions (R5-tropic) for 3 h, and two-color images of the amyloid fibers (red) and HIV-1 virions (green) were created using fluorescence confocal microscopy. As shown in Fig 5A, regardless of the presence of EP2, PAP248-286 amyloid fibrils could aggregate HIV-1 virions at different time points following agitation. Furthermore, the presence of amyloid fibrils enhanced the attachment of HIV-1 virions to target cells, as verified by three-color imaging of the fibrils (red), virions (green), and cells (blue; Fig 5B). Taken together, these data suggest that, regardless of the presence of EP2, PAP248-286 amyloid fibrils promote interactions beween HIV-1 virions and target cells.

### PAP248-286 amyloid fibrils formed in the presence of EP2 retained the ability to enhance HIV-1 infection

**Fig 6:**
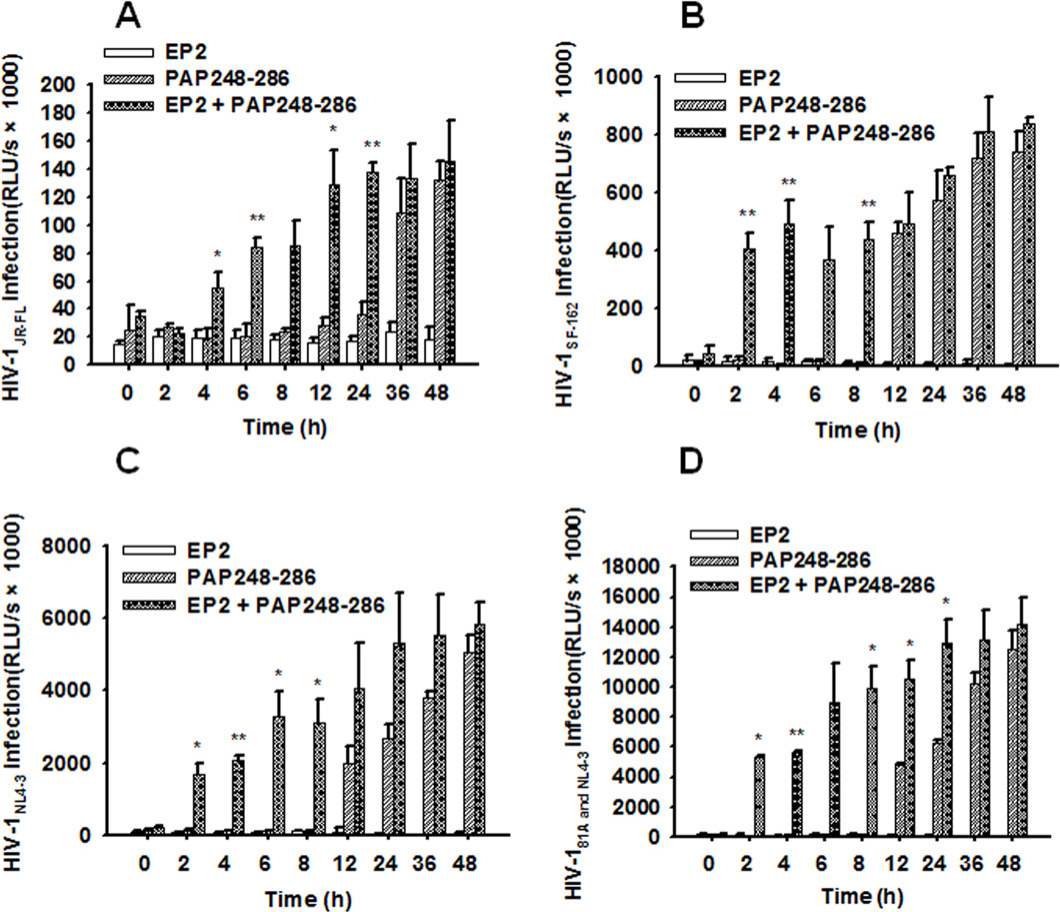
EP2 promoted PAP248-286 amyloid fibril formation and thereby enhanced HIV infection. Fibrils were generated by incubating 3 mg/ml PAP248-286 in the presence or absence of EP2 (100 μg/ml). At the indicated time points, (A) the addition of fibril samples (30 μg/ml) increased the infectivity of R5-tropic HIV-1_JR-FL_ Env-pseudotyped viruses in target cells. The fibril samples promoted the infectivity of different infectious HIV clones, including R5-tropic (B), X4-tropic (C) and dual-tropic infectious HIV-1 clones (D). The means (± standard deviations) of triplicate samples are shown. RLU/s, relative light units/second. One-way ANOVA and Dunnett’s post hoc multiple comparisons test were used to statistically analyze differences between the group of viruses treated with PAP248-286 amyloid fibrils and the group of viruses treated with amyloid fibrils plus EP2 (*p<0.05; **p<0.01).

Based on the above findings, we utilized an infection assay to investigate whether PAP248-286 amyloid fibrils formed in the presence of EP2 could enhance HIV-1 infection. In the absence of EP2, only PAP248-286 amyloid fibrils collected after 36 to 48 h of shaking at 37°C could increase the infectivity of R5-tropic HIV-1 Env-pseudotyped viruses on U87-CD4-CCR5 cells. No enhancement was observed if the amyloid fibrils were collected prior to 24 h of agitation (Fig 6A). Conversely, PAP248-286 amyloid fibrils formed in the presence of EP2 significantly enhanced HIV-1 infectivity after shaking for only 2 to 4 h at 37°C (Fig 6A). This finding may be attributed to the accelerated formation of PAP248-286 amyloid fibrils mediated by EP2. The infectivity-enhancing properties of PAP248-286 amyloid fibrils formed in the presence or absence of EP2 were also tested in TZM-bl cells infected with R5-tropic, X4-tropic and dual-tropic infectious HIV-1 clones. As expected, similar results were observed when using amyloid fibrils formed after 2 h of agitation in the presence of EP2; these fibrils exhibited a progressively increasing ability to enhance the infectivity of the different infectious HIV-1 clones (Fig 6B–6D). PAP248-286 amyloid fibrils formed in the absence of EP2 also possessed the ability to enhance HIV infectivity after shaking for 12 to 48 h (Fig 6B–6D).

## Discussion

Seminal plasma proteins, such as PAP fragments (e.g., PAP248-286), or bacterial curli proteins may promote the formation of seminal amyloid fibrils, which in turn dramatically enhance HIV-1 infection. These amyloid fibrils in semen are considered an endogenous enhancer of viral infection and are important for sexual transmission of HIV-1 [10, 30, 34]. Here, we reported the details of the interaction between a short peptide derived from the HIV-1 gp120 coreceptor-binding region (EP2) and seminal amyloid fibrils.

Numerous envelope glycoprotein complexes disassemble when HIV-1 particles reach target cell surfaces; this disassembly results in the release of gp120 [35]. Lysosomal or ubiquitin-mediated intracellular degradation pathways play a vital role in the degradation of HIV-1 envelope proteins [24, 36]. Several studies have demonstrated that gp120 is internalized by binding to either CD4/CCR5 or to the mannose 6-phosphate receptor (MPR) and that internalized gp120 is cleaved into small peptides or amino acids in the cytoplasm by lysosome/ubiquitin enzymes [36, 37]. Gp120 is also degraded by catalytic antibodies through a nucleophilic mechanism due to host immune responses [23, 38, 39]. Indeed, it is critical to determine whether EP2 seeds exist in the human body during HIV transmission. Interestingly, residues 421–433 (KQIINMWQEVGK) are located in the B cell SAg site of gp120; immunoglobulins, such as IgMs, IgAs and isolated L chain subunits, hydrolyze the peptide bonds located within this epitope via a nucleophilic mechanism [23]. This vulnerability of the 421–433 epitope can induce the production of powerful neutralizing IgA antibodies in long-term survivors of HIV infection [40]. Following the degradation of gp120, several short peptide fragments, including INMWQG, QVFYRTGD and RTGDIIGDIRK, have been found in native gp120-loaded rat hepatocytes [24]. Notably, using liquid chromatography-mass spectrometry/mass spectrometry analysis (LC-MS/MS), our groups have found that the sequences of some fragments in HIV-1 culture supernatants matched those of previously reported short EPs derived from gp120 (data not shown). More extensive studies of HIV-infected patients will be conducted in the future. Collectively, the present data indicate that biologically relevant EP2 analogs might be present in nature.

Many different types of soluble proteins and peptides can form amyloid fibers. These fibers are insoluble, highly ordered, extremely strong and resistant to degradation [41]. In general, amyloid fibril formation occurs in the following three stages: (i) monomers are converted into small oligomers in entropically unfavorable folded conformations, (ii) fibrils grow after nucleation, and (iii) fibrils rapidly aggregate until equilibrium is reached [42]. As we previously demonstrated, at high concentrations, select EPs (up to 1 mg/ml) can assemble into amyloid fibrils to enhance HIV infectivity [21]. Notably, EP2, a 15-residue peptide derived from the HIV-1_MN_ gp120 coreceptor-binding region (QCKIKQIINMWQEVG), contains the short peptide fragment INMWQG, which is a degradation product of gp120. Furthermore, the sequence of EP2 also overlaps with residues 421–433 (KQIINMWQEVGK), which can be hydrolyzed by Ig. In the present study, we investigated the effects of EP2 on seminal amyloid fibril-mediated enhancement of HIV-1 infection.

Amyloid fibrils are polypeptide-based polymers or highly organized protein filaments that vary in size (commonly at the low micrometer level) and in formation kinetics. Recently, it has been discovered that a subset of short peptides can undergo spontaneous assembly into ordered nanofibrils [19, 43]. Nanofibrils are nanoscale amyloid fibrils. Evidence suggests that nanofibrils and amyloid fibrils differ both in morphology and length [19, 28, 44]. Here, we showed that EP2 can very rapidly form fibrils by self-aggregation following dilution into PBS at room temperature (Fig 1A and 1B). Analysis of the mean hydrodynamic size and zeta potential of the EP2 fibrils suggested that these fibrils are indeed nanofibrils (Fig 1C and 1D); these results are consistent with a previous report by Yolamanova et al. [19]. Zeta potential is associated with the degree of electrostatic repulsion in a dispersion. At low zeta potential (from 0 to 30 mV), attractive forces may exceed this repulsion, and the dispersion may break and flocculate [45, 46]. We found that EP2 fibers have a zeta potential of +16.7±1.0 mV, which suggests that EP2 in solution has a tendency to aggregate. Furthermore, the positive zeta potential of EP2 fibers may neutralize the negative charge repulsion that exists between HIV virions and target cells [11]. Nanofiber formation was further confirmed by TEM. Compared with SEVI amyloid fibrils (typically straight and rod-like), EP2 nanofibers are shorter and more malleable (Fig 1E and 1F).

Theoretically, PAP fragments, such as PAP248-286, do not enhance HIV-1 infection when in monomeric form. Therefore, to act as SEVI amyloid fibrils, PAP fragments must form amyloid fibrils. This formation is controlled by the rate of amyloidogenesis. However, the creation of SEVI amyloid fibrils is challenging due to the stochastic nature of the amyloid assembly process. We further investigated whether the presence of EP2 nanofibers can accelerate the formation of PAP248-286 (a fragment of PAP that is present in human semen) amyloid fibrils. As shown in Fig 3, the addition of a very low concentration of preformed EP2 fibrils to a solution of PAP248-286 dramatically reduced the lag time of amyloid fibril formation (approximately 2 h) and therefore significantly increased the rate of amyloid fibril formation, as measured by both ThT fluorescence and Congo red assays. TEM results further confirmed the above conclusions (Fig 2). Therefore, EP2, which is derived from the HIV-1 envelope protein gp120, accelarates the formation of semen amyloid fibrils by PAP fragments, such as PAP248-286.

We next determined whether PAP248-286 amyloid fibrils formed in the presence of EP2 enhanced HIV-1 infectivity in a similar manner to semen amyloid fibrils generated *de novo*. As shown in Fig 6, PAP248-286 amyloid fibrils formed in the presence of EP2 exhibited an increased ability to enhance HIV-1 infection by R5-tropic, X4-tropic and dual-tropic infectious clones compared to amyloid fibrils formed in the absence of EP2.

The PAP248-286 monomer lacks significant ordered secondary structure. The cross-β-sheet structure common to all amyloid fibrils is created by intermolecular associations of β-sheets that are stabilized by hydrogen bonds [15, 16]. Similar to amyloid fibrils formed in the absence of EP2, those formed in the presence of EP2 exhibited an obvious transition from a random coil structure to a β-sheet formation after shaking for 8 h at 37°C. These results suggest that EP2 can promote the transition of PAP248-286 from a random coil structure into a β-sheet conformation (Fig 4).

Previous studies have shown that the natural positive charge of PAP248-286 amyloid fibrils (pI = 10.21) facilitates the attachment of HIV-1 virions to target cells. Thus, these amyloid fibrils enhance viral infection by neutralizing the charge repulsion that otherwise exists between HIV-1 virions and host cells [11]. Next, we utilized fluorescence confocal microscopy to investigate whether EP2 nanofibers (the theoretical pI value of EP2 is 8.20) and PAP248-286 amyloid fibrils formed in the presence or absence of EP2 affect the binding of HIV-1 virions to the surfaces of host cells. We found that, in the absence of EP2, PAP248-286 amyloid fibrils could enhance the binding of HIV-1 virions to target cells after shaking for 48 h. However, PAP248-286 amyloid fibrils formed in the presence of EP2 enhanced the binding of HIV-1 virions to target cells after shaking for only 8 h (Fig 5).

**Fig 7:**
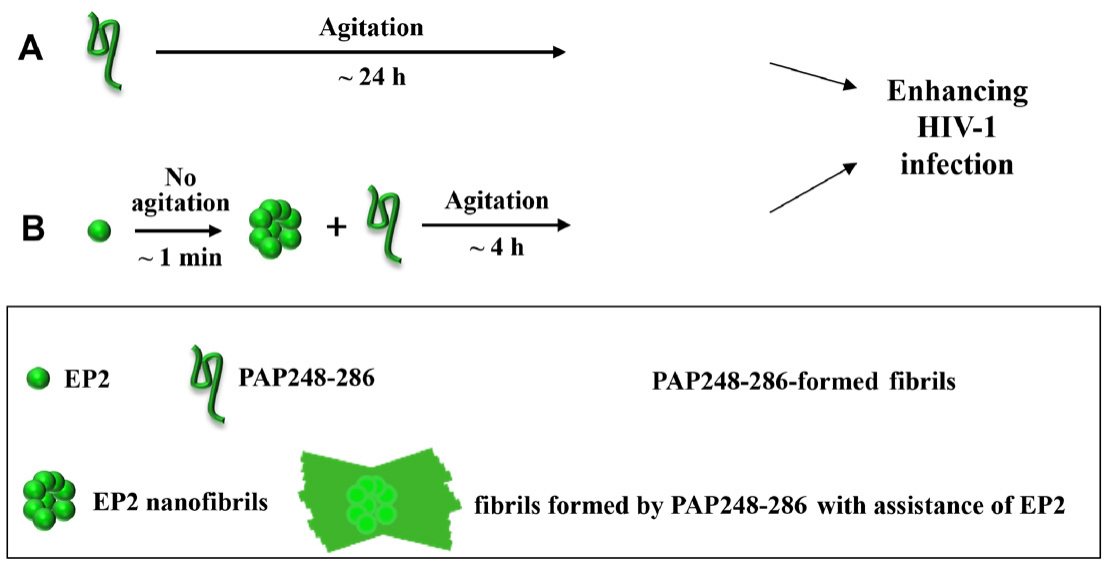
Schematic representation of EP2’s mechanism in promoting PAP248-286 amyloid fibril formation and enhancing HIV-1 infection. (A) PAP248-286 amyloid fibrils enhanced HIV-1 infection. Under agitation at 37°C, PAP248-286 slowly (~ 24 h) formed amyloid fibrils, which enhanced HIV-1 infection. (B) EP2 promoted the formation of PAP248-286 amyloid fibrils. Without agitation, EP2 rapidly (~ 1 min) self-assembled into nanofibers. These nanofibers accelerated (~ 4 h) the formation of amyloid fibrils, which enhanced HIV-1 infection.

In summary, EP2, a 15-residue peptide derived from the HIV-1 gp120 coreceptor-binding region, can self-assemble into nanofibers and accelerate the formation of amyloid fibrils by PAP fragments, such as PAP248-286, in semen. These newly formed amyloid fibrils enhance HIV-1 infection by promoting the binding of HIV-1 virions to target cells. The mechanism underlying how EP2 seeds drive PAP248-286 amyloid formation and thereby enhance HIV-1 infection is detailed in Fig 7. Therefore, analogs of EP2 might be useful for the development of novel HIV-1 entry inhibitors.
