# Correction: A Peptide Derived from the HIV-1 gp120 Coreceptor-Binding Region Promotes Formation of PAP248-286 Amyloid Fibrils to Enhance HIV-1 Infection

**DOI:** 10.1371/journal.pone.0147679

**Published:** 2016-01-27

**Authors:** Jinquan Chen, Ruxia Ren, Suiyi Tan, Wanyue Zhang, Xuanxuan Zhang, Fei Yu, Tianrong Xun, Shibo Jiang, Shuwen Liu, Lin Li

Fig 7 is incorrect. The figure is incomplete. The authors have provided a new, completed version here.

**Fig 7:**
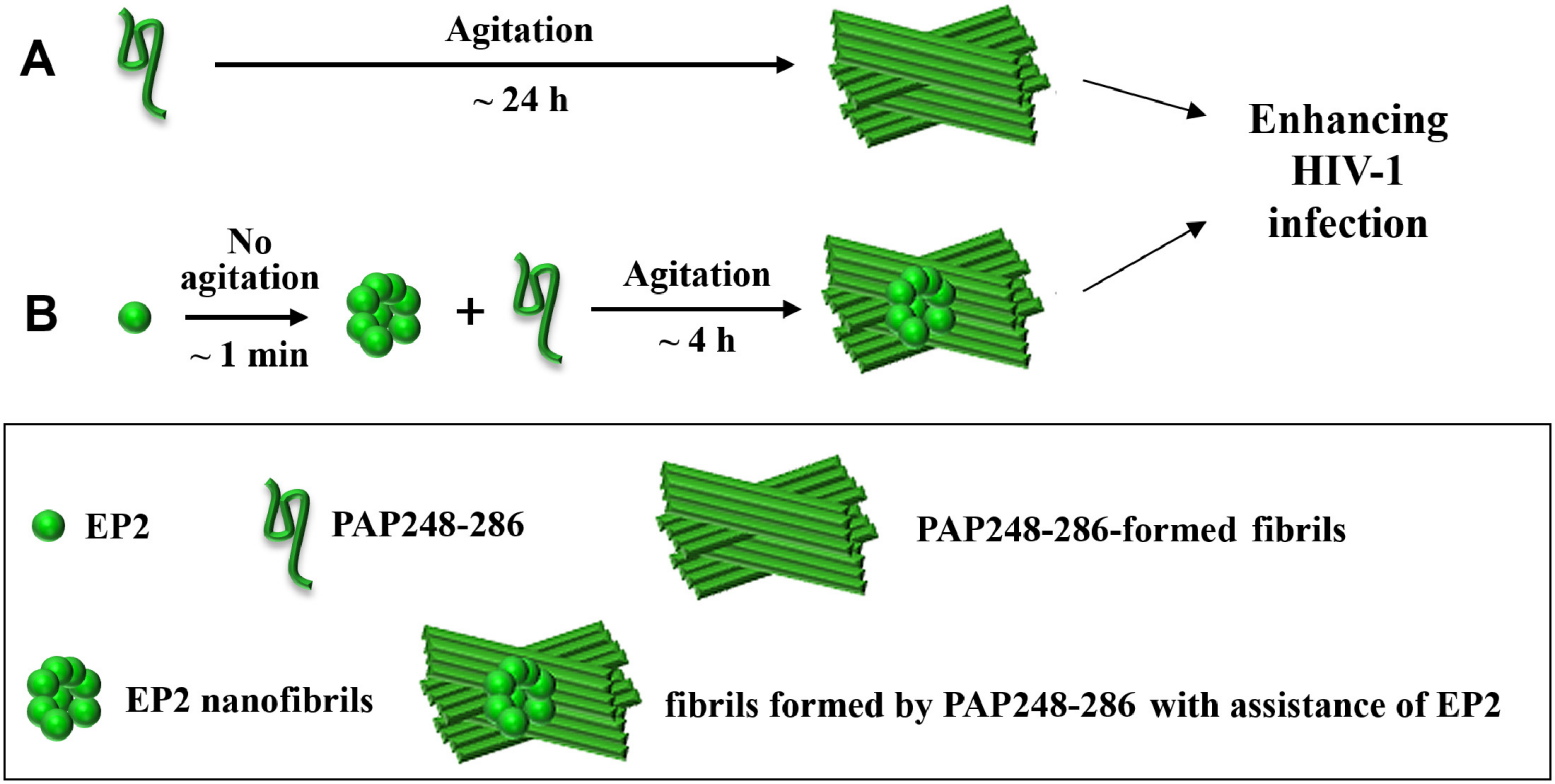
Schematic representation of EP2’s mechanism in promoting PAP248-286 amyloid fibril formation and enhancing HIV-1 infection. (A) PAP248-286 amyloid fibrils enhanced HIV-1 infection. Under agitation at 37°C, PAP248-286 slowly (~ 24 h) formed amyloid fibrils, which enhanced HIV-1 infection. (B) EP2 promoted the formation of PAP248-286 amyloid fibrils. Without agitation, EP2 rapidly (~ 1 min) self-assembled into nanofibers. These nanofibers accelerated (~ 4 h) the formation of amyloid fibrils, which enhanced HIV-1 infection.
